# An evaluation of online information acquisition in US news deserts

**DOI:** 10.1038/s41598-024-77303-y

**Published:** 2024-11-13

**Authors:** Kevin T. Greene, Nilima Pisharody, Alonso Guevara, Nathan Evans, Jacob N. Shapiro

**Affiliations:** 1 Princeton University, Princeton, USA; 2 New York University, New York, USA; 3 Microsoft Research, Microsoft, Redmond, USA

**Keywords:** Information technology, Computational science

## Abstract

A growing concern is that as local newspapers disappear, communities lose trusted gatekeepers and develop information voids, creating openings for misinformation to thrive. Previous work has not evaluated whether residents of news deserts have developed different information acquisition habits. We fill this gap by directly comparing information consumption and referral patterns inside and outside of news deserts in a novel dataset of engagement with online media by millions of users on the Edge browser. We find little evidence that those in news deserts consume more low-quality sites or are more likely to be referred to low-quality sites from search engines or social media. We find some evidence that those in news deserts do consume more national news than locations with local media outlets. These results contribute to our understanding of how the loss of local newspapers has impacted online information acquisition.

## Introduction

Between 2005 and 2020, more than 25% of U.S. newspapers ceased operations, leaving roughly half of U.S. counties with only a single paper, and 200 with no paper at all^1,2^. As local papers are the most trusted information sources in the U.S.^3,4^ and fill most of the “critical information needs” (e.g. health, education, emergencies)^5,6^, their loss has been linked to decreased civic engagement, lower political knowledge, and less accountability for elected officials^1,7–11^. These negative impacts have led some to claim the growth of *news deserts* (i.e. locations without local newspapers) represent an ongoing threat to democracy^12–14^.

The diminished local information environments in the U.S. are currently faced with another threat, the proliferation of online misinformation. The loss of local media leaves both an information gap and the loss of a bulwark against misleading information^4,15–17^. Many suggest this creates openings for misinformation to thrive^4,12–18^, while leaving locations more vulnerable to exposure to misinformation from social media and search engines^13,16,19^. However, the notion that news deserts are being overrun with misinformation has not been tested at scale. We currently know little about differences in media consumption between news deserts and other places, or if people in news deserts are more likely to be pushed to low-quality information sources.

In this paper, we examine the relationship between news deserts and information acquisition habits, focusing on (a) the types of sites consumed and (b) the types of sites directing traffic to low-quality news sites which fail to meet core reporting and transparency standards. Such sites contain more misinformation^20^ and social media posts linking to them are more likely to contain false claims^21^. We measure these quantities by combining data on the locations of news deserts^2^ with telemetry data that capture the browsing activity of millions of U.S. users on the Microsoft Edge browser from August to September 2021. The browsing data are anonymized and aggregated to remove all personal or identifying information and collected from opt-in users. These data allow us to measure county-level engagement with low-quality news sites, as well as other information sources. Our data also captures the sites leading to low-quality sites through referrals, allowing us to evaluate the routes taken to them.

Our results contrast with previous expectations that misinformation would flourish in news deserts in two respects. First, we find little evidence that people in news deserts consume more low-quality sites or pink slime (domains publishing partisan content or corporate P.R. while masquerading as local media)^22^ than those in locations with stronger local media. While many have worried that the void created by the loss of local media would be filled with misinformation, the browsing data do not show that low-quality sources are consumed at a higher rate in locations without local newspapers. Second, despite worries that those in news deserts are more likely to be directed to low-quality sites by social media or search engines^13,16,19,23^ we find no evidence that this is the case. Finally, we find some evidence that news deserts consume more national news than locations with local media outlets. However, as national news contain less critical information and more intense partisan cues^24–26^, this may be a poor substitute for local media.

The next section reviews arguments for why information acquisition habits in news deserts would be different. The Materials and Methods section describes our research design, including our online browsing data and categorization of information sources.

### Information acquisition in news deserts

A key proposed mechanism through which news deserts impact communities is by changing the type of content that is consumed (information acquisition habits)^24,25^. Without local papers, there is an information gap, and the loss of a highly trusted information source^3,4,15–17^. With local news no longer available, this void is thought to be filled by other, lower-quality sources of information, including sites that spread misinformation^12,13,16,18^. However, this relationship has often been assumed rather than tested^10^. In the following sections, we draw from previous literature to specify two research questions on information acquisition strategies. While previous work has made compelling arguments for why the consumption habits in news deserts may differ from locations with local media, these arguments have not been directly tested at scale.

Previous work has proposed that news deserts consume different content than locations with robust local media. Without local papers, people turn to other information sources. This leads to our first research question RQ1: *Do people in news deserts consume different kinds of news than locations with local newspapers?*. To answer this question we examine the consumption of low-quality news, ‘pink slime’ sites that masquerade as local news, and national news.

#### Low-quality sites

Many in academia and the popular media have suggested that the gap left by the disappearance of local media will be filled by outlets that fail to meet journalistic standards and distribute misinformation^12,13,16,18^. Past work suggests that in news deserts, misinformation has impacted public trust in government^15^ and may have led to additional deaths during the COVID-19 pandemic as misinformation was better able to take hold^17,19^.

#### Pink slime

Others argue that the paucity of local newspapers may also lead to increased engagement with websites masquerading as local news sites. These so-called ‘pink slime’ sites, named after the meat by-product used to cheaply imitate higher quality products, often have the appearance of a local paper but do not hold similar journalistic standards. These sources leverage the high trust in local news to push ideologically biased content^3^ and misinformation^27–30^. Pink slime sites are often part of large networks relying on algorithmically generated content^31^ and often have undisclosed ties to political parties^15,32,33^.

#### National news

Finally, some suggest the void left by failed local newspapers may be filled by national media outlets. While many communities lack local papers, media outlets such as *CNN* or national papers with a strong online presence are more widely available. More national news likely means decreased coverage of local issues^34^, increased partisan heuristics, and more focus on horserace politics, rather than information provision^24–26^.

### Differences in referrals to low-quality sites

Others have suggested that the loss of local papers may have also reshaped how users arrive at low-quality sites^16,23^. This leads to our second research question RQ2: *Do news deserts arrive at low-quality sites through different channels than locations with local newspapers?*.

#### Search engines/social media

Locations without local media are expected to consume more information online where there are fewer gatekeepers^23^ while having fewer opportunities to receive trusted local information^13,16,19^. This leaves news deserts more susceptible to being led to misinformation by social media and search engines. Previous work^20,35–38^ has consistently found that social media and search engines are the most prominent referrers to misinformation. But we are unaware of evidence that those in news deserts are more likely than others to be referred to low-quality sites from these sources.

## Results

**Fig. 1:**
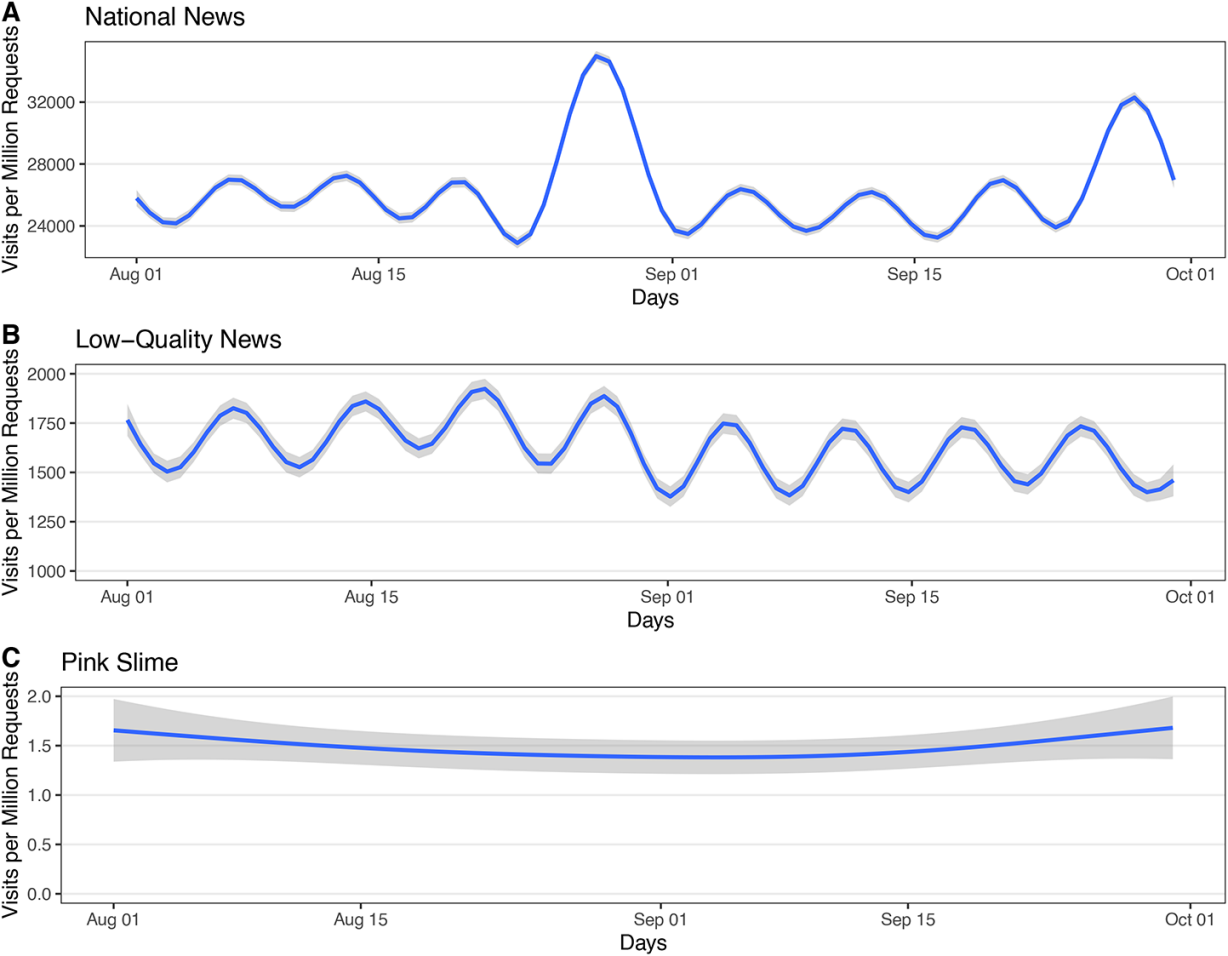
Average daily visits per million requests by content type: (**A**) national news sites; (**B**) low-quality news sites; and (**C**) pink slime sites. Each plot includes the mean over counties (blue) and a corresponding 95% confidence interval (gray). The unit of analysis is the county-day. For each content type, requests are normalized by the total requests for the county day and then multiplied by one million.

As noted in previous work^39^, news media make up a relatively small proportion of overall web activity (Figure 1). Further, views for national news are over an order of magnitude larger than low-quality news, while pink slime sites appear to receive almost no traffic. We also observe clear periodicity in both views of national news and low-quality news. Views for both sources fall on the weekends. The large spikes in views for national news align with the U.S. withdrawal from Afghanistan, and Pfizer’s submission of results of their COVID-19 vaccine for children.

### Consumption patterns

**Fig. 2:**
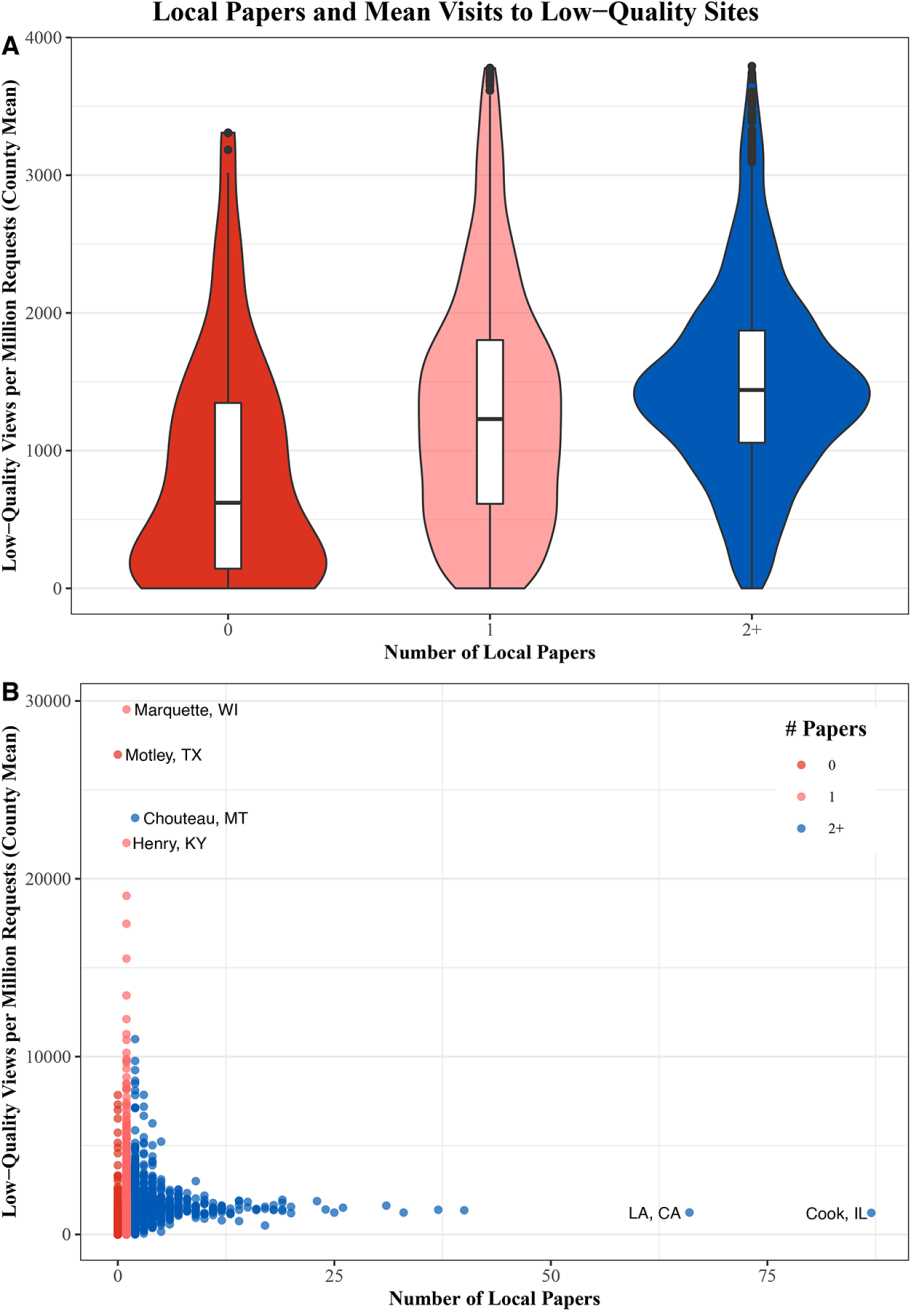
Local papers and consumption of low-quality content. (**A**) Distribution of low-quality news site views per million requests at the county level for locations with no local papers, one local paper, and more than one local paper. (**B**) The mean level of low-quality content views and the number of local papers (including weekly papers) for each county in the U.S. The colors of the points correspond to the location types in (**A**). Counties with especially high consumption of low-quality content and those with a large number of local papers are highlighted. The unit of analysis is the county. For each content type, requests are normalized by total requests for the county and then multiplied by one million.

Contrary to previous expectations, we see little evidence that news deserts view more low-quality content than locations with local newspapers (Figure 2 A). In fact, news deserts appear to view less low-quality information per million requests (median=721) than locations with two or more local papers (median=1461). Note, because these numbers are scaled by the number of requests, they do not simply reflect the fact that more populous counties are more likely to have local newspapers. Further, the amount of low-quality information viewed in locations with a single paper and locations with multiple papers are similar (median=1302 vs. median=1461). Of the ten counties that view the most low-quality information, only a single county has no local newspaper (Figure 2 B).

**Fig. 3:**
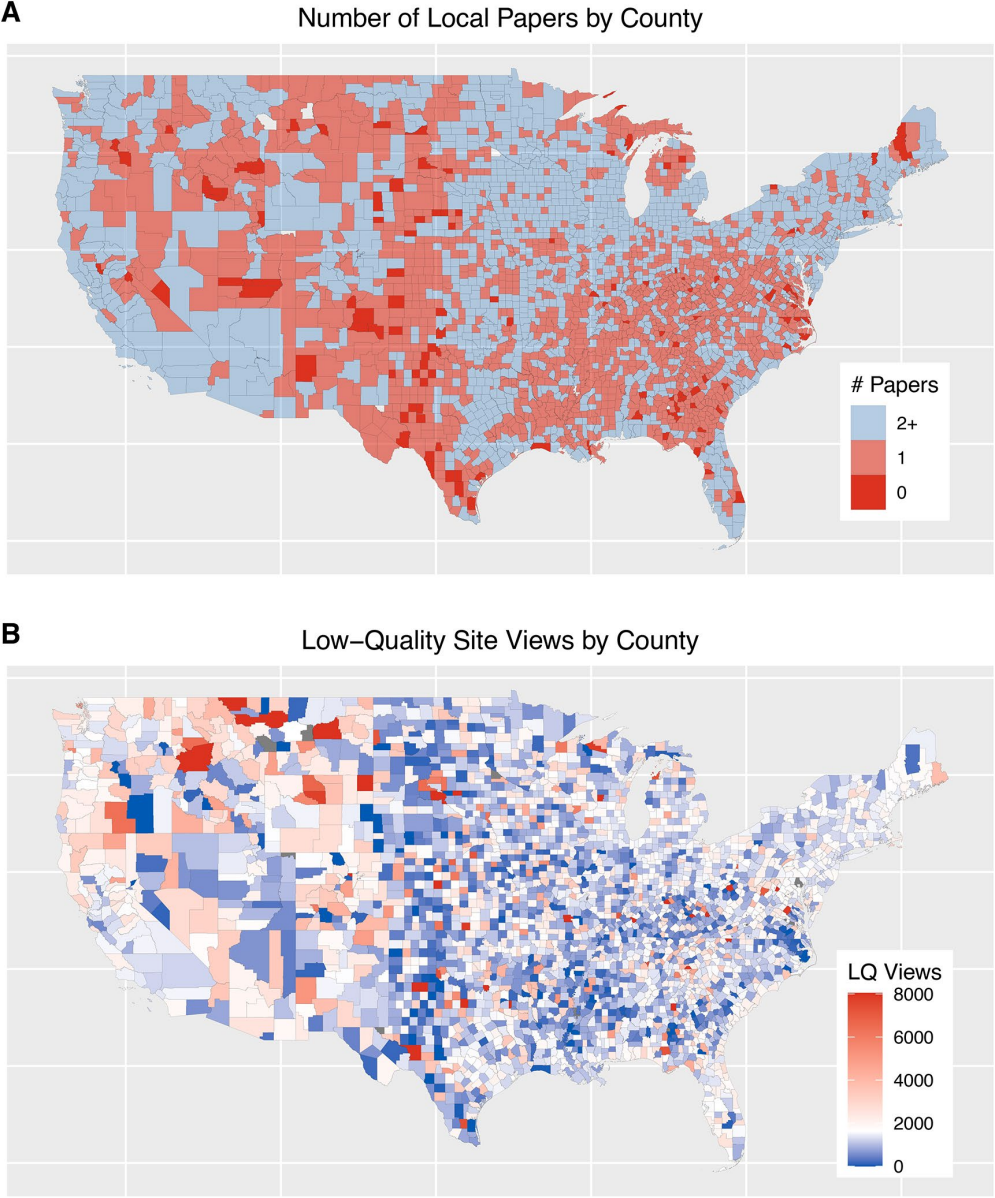
The geographic distribution of news deserts and low-quality views. (**A**) The number of local papers in each county. Counties with no papers are colored red, counties with a single paper are light red, and counties with more than one paper are blue. (**B**) The amount of low-quality sites viewed (per one million requests). Locations with views of low-quality information near the national mean are white. Locations viewing less than the mean are colored blue, while counties viewing more than the mean are colored red. The figure was generated using R v3.6.3.

To provide additional context, we present the geographic distribution of news deserts (Figure 3 A) and low-quality information consumption (Figure 3 B). First, as noted by^1,2^, the loss of local media is a large-scale problem, impacting every state in the country. The loss of local news also appears to be particularly severe in the South and Southwest. In Figure 3 B, for each county, we use a count of the number of views of low-quality sites (per one million total requests). The color scale is centered on the national average amount of low-quality site views. Counties that view less low-quality information than the national average are colored blue, while counties that view more low-quality information are colored red. Counties whose views of low-quality information are at least four standard deviations greater than the mean are colored dark red. We do not observe any clear patterns between news deserts and the locations that view the most low-quality information. For example, the counties near the Idaho-Montana border appear to view a considerably higher than average amount of low-quality information, but these areas generally have multiple local papers. In contrast, many counties in the Southeast have few local papers but view relatively little low-quality information.

To further evaluate RQ1, we estimate a series of linear regressions. Our outcome variables measure views per one million requests of either national news sites, low-quality news sites, or pink slime sites. Our key independent variable is a binary indicator for whether a given county is a news desert^2^. In the Supplementary Materials, we conduct additional analyses with varying definitions of news deserts, finding consistent results. For each outcome, we estimate three models. The first model (M1) captures the bivariate relationship between news deserts and each of our outcome variables. The second model (M2) controls for county-level demographics and uses coarsened exact matching^40,41^ to reduce the imbalance between locations with and without local newspapers. The third model (M3) includes the same control variables in model (M2) as well as state and day fixed-effects to account for unobserved between-unit heterogeneity and differences over time. This model does not include matching. Across models, standard errors are clustered on the state-month. In the Supplementary Materials, we report models with the standard errors clustered on the state and reach similar conclusions. A growing body of literature finds that the ownership of local television stations impacts political attitudes and information consumption^34,42^. We account for this by controlling for the presence of local television stations owned by the Sinclair Broadcast Group. We report these models in the Supplementary Materials and reach similar conclusions.

**Fig. 4:**
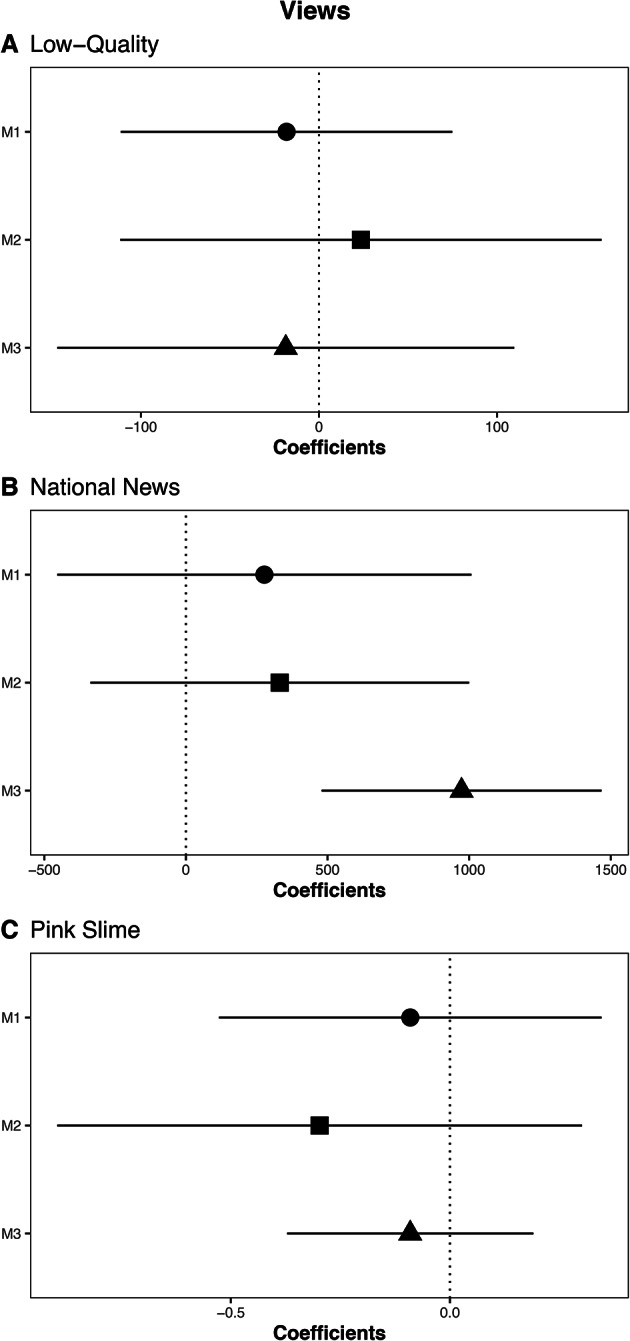
Coefficients and 95% confidence intervals for the impact of news deserts on consumption habits. (**A**) views of low-quality news (**B**) views of national news (**C**) views of pink slime. In each figure M1 indicates the bivariate correlation, M2 includes control variables and coarsened exact matching, and M3 includes control variables and fixed effects. We control for county level: population, median household income, % with broadband internet, % white, % with a college degree, and Republican vote share. Standard errors are clustered on the state-month.

Two key findings emerge. First, contrary to previous expectations, we find no statistically significant evidence that news deserts consume more low-quality news site content than locations with more robust local media (Figure 4, A). This is true when looking at the bivariate correlation (M1, $\beta$=-18.22, *SE*=47.50), after applying coarsened exact matching (M2, $\beta$=23.55, *SE*=68.95), and after including state and day fixed effects (M3, $\beta$=-18.66, *SE*=65.47). We observe similar results for pink slime sites (Figure 4, C). Across models news deserts are associated with viewing less pink slime content. However, these results are not statistically significant at conventional levels. M1 ($\beta$=-0.09, *SE*=0.22), M2 ($\beta$=-0.297, *SE*=0.305) and M3 ($\beta$=-0.09, *SE*=0.143).

Second, we find some evidence that those in news deserts consume more national news than those in locations with more robust local media (Figure 4, B). Across models, we observe that news deserts are related to increased views of national news. This is true for M1 ($\beta$=276.90, *SE*=373.04), M2 ($\beta$=331.21, *SE*=341.161), and M3 ($\beta$=973.28, *SE*=252.18). However, this is only statistically significant for M3. In practical terms, the difference in consumption of national news between news deserts and non-news deserts is roughly equal to the difference in news consumption (normalized by total requests) between Philadelphia and New York. When we use a stricter definition of news deserts, we still see no evidence that news deserts are associated with viewing more low-quality content. These results are presented in the Supplementary Materials.

Among our other control variables, only county population is consistently associated with views of low-quality news domains. Locations with smaller populations consume a larger proportion of low-quality domains. This differs from past work finding that Republicans consume more low-quality sources^43–45^. This discrepancy is likely explained by past work using individual-level data, while our data is aggregated at the county level.

### Referral patterns to low-quality sites

To evaluate our second research question, we again estimate a series of linear regressions. We estimate the same models as our previous analyses, changing the outcome variables to measure the number of views of low-quality sites that were referred to by either social media or search engines per one million referrals. Our key independent variable is a binary indicator that records if a given county is a news desert^2^.

**Fig. 5:**
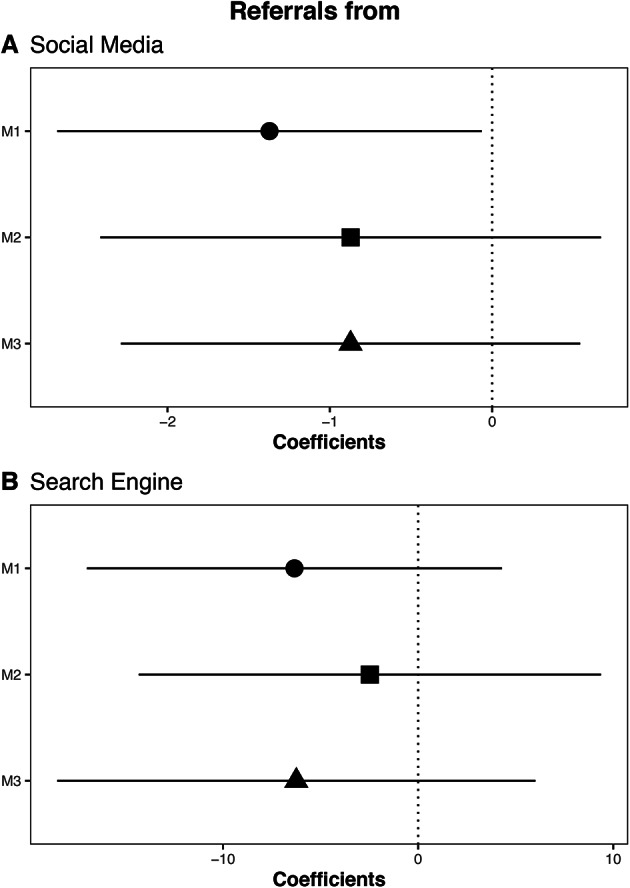
Coefficients and 95% confidence intervals for the impact of news deserts on referrals to low-quality sites sites. (**A**) referrals from social media (**B**) referrals from search engines. In each figure M1 indicates the bivariate correlation, M2 includes control variables and coarsened exact matching, and M3 includes control variables and fixed effects. We control for county level: population, median household income, % with broadband internet, % white, % with a college degree, and Republican vote share. Standard errors are clustered on the state-month.

Our results provide little evidence that news deserts are more likely to be referred to low-quality sites by social media (Figure 5, A). For M1 ($\beta$=-1.37, *SE*=0.67) we see fewer referrals to low-quality sites from social media for news deserts. For models M2 and M3, we again see fewer referrals from social media, but these results are not statistically significant. Our results also provide little evidence that news deserts are more likely to be referred to low-quality sites by search engines (Figure 5, B). In all three models news deserts are associated with fewer referrals from search engines. However, this relationship is not statistically significant at conventional levels. Taken together we find no evidence that that referrals to low-quality sites are more likely in news deserts.

## Discussion

We evaluate whether news deserts display different patterns of consumption and referral patterns from locations with local newspapers. We find little evidence that the vacuum left by the collapse of the local newspaper industry has been filled by low-quality sites. News deserts do not consume more information from either low-quality sites or pink slime sites. Our findings are consistent with recent results that find null effects for individuals taking short-term “news vacations”^46^. Further, we find no evidence that news deserts are more likely to be referred to low-quality sites from either social media or search engines. However, we do find some evidence that news deserts consume more national news than locations with local media outlets. These results are consistent across a variety of measures of news deserts.

These results suggest that fears of rampant misinformation in news deserts may be overstated and present a more optimistic case for efforts aiming to bolster local journalism. Rather than having to struggle against news diets filled with misinformation, it appears that an important task is filling the critical information gaps left after the disappearance of local papers. Further, as theorized by^24^, our results also suggest that the importance of local newspapers is to balance out other news sources. If news deserts consume the same amount of low-quality sites but do not have a trusted local paper to provide higher-quality information, the public can still be misinformed. This balancing may be particularly important because we find some evidence that news deserts consume more national media, which tends to be filled with stronger partisan cues^24^.

Our results have some limitations. First, our measure of news deserts does not vary over time or provide information about how long a location has been a news desert. Future work might use an approach suggested by^47^ to construct a more dynamic measure. Second, we evaluate the correlation between the presence of local newspapers and online information consumption, we are not able to make causal claims based on this data. Further, preexisting consumption preferences may have worsened the decline of the local newspaper industry. Third, while search and social media referrals are based on algorithms that are constantly evolving, our study necessarily captures a fixed time period of referral patterns. Additional changes to these systems could impact the findings. In particular, it would be useful for future work to evaluate changes that may plausibly have differential impacts in locations with and without local papers. Fourth, a common limitation of desktop browser-based studies of information consumption habits^20,45,48^ is they are unlikely to be representative of all Americans. In particular, desktop browser activity tends to skew towards older individuals^49^. Thus our findings may not hold for younger Americans. Fifth, to ensure our results are not driven by a single organization’s criteria for low-quality sites we use the intersection of domains contained in list compiled by multiple raters. However, given the high correlation between different sets of ratings^50^ a less conservative approach would be to use the union of domains across raters. Sixth, in the absence of an authoritative list of national media domains, we used a data-driven approach to select these sites, relying on the 25 news domains that received the most web traffic in the United States. Alternative criteria for national news may produce different results. Including additional features of these news sites would allow further assessments of patterns in consumption between locations with and without local papers. As a precaution in the Supplementary Information we conduct a set of analyses with a varying set of domains and find consistent results. Finally, while we believe this is the largest study of the information acquisition strategies within news deserts, it is purely within the United States. It would be valuable for future efforts to evaluate whether these findings hold outside the United States because the decline of local media is not a uniquely American phenomenon. In particular, others have pointed out that the formation of news deserts in Brazil may be particularly severe^13^.

While we do not find that news deserts consume more low-quality sites, our results cannot rule out other harmful impacts of low-quality sites in news deserts. For instance, it might be that individuals that reside in news deserts are less discerning about false information or more resistant to fact-checking. Extensions of the work by^48,51^ could directly test this theory. Further, differences in information acquisition habits might be starker during election years. Building on^20^, efforts to measure the browsing behavior of individuals during the 2024 U.S. election could incorporate information about the strength of local media to further evaluate the findings presented here.

## Materials and methods

### National media

In the absence of an authoritative list of national media domains, we use a data-driven approach to identify national media sites. In particular, we select the top twenty-five news sites in the U.S. based on overall traffic. Given the high concentration of traffic on a few sites^52^ this lists capture sites that represent much of the total traffic. The data on web traffic is from Similarweb and was recorded from October to December 2020. The full list of sites includes: cnn, nytimes, foxnews, msn, news.google, washingtonpost, finance.yahoo, usatoday, cnbc, news.yahoo, nypost, bbc, dailymail, politico, nbcnews, theguardian, businessinsider, thehill, huffpost, forbes, people, wsj, cbsnews, yahoo, abcnews. In the Supplementary Information we rerun our analyses excluding three non-US based domains. There is one common site between our lists of national media and low-quality sites, *The Daily Mail*.

### Low-quality sites

Previous studies use different terms to describe unreliable news domains (e.g., misinformation, fake news). We use the term low-quality to encompass sites that have been rated as poor information sources by academic or fact-checking groups. We construct our list of low-quality information sources by consulting a variety of existing work on the subject. In total, we use four sources that maintain lists of low-quality sites: three lists of domains complied by academic researchers^53–55^; and one from a fact-checking and media accountability groups (Media Bias/Fact Check). For our analysis, we consider a site to be low-quality if it has been identified by at least two of the sources. This approach ensures that our results are not driven by a single organization’s criteria for low-quality sites. Our final list contains more than 1300 low-quality domains in total. In the Supplementary Information we rerun our results after removing Bitchute from our list of low-quality domains, as it is a video sharing platform that differs significantly from other low-quality news sources^56^.

### Pink slime

Our measure of pink slime journalism sites comes from investigations carried out by the Columbia Journalism Review and the New York Times^22,27,57^. They uncovered a network of seemingly local papers operating across the United States. The data contains nearly 1300 domains in total.

### News desert data

the data on news deserts in the United States is drawn from^2^. The data provides a count of the number of local papers in each county in the United States. Using this data, we construct three measures of news deserts. First, we code a county as a news desert if there are no local papers in the county. Second, we code a county as a news desert if it has at most one local paper. Third, we code a county as a news desert if it has no local paper released daily.

### Browsing data

We collected two months of telemetry data from the desktop browser, Microsoft Edge. This data was collected from users who had opted to share diagnostic information. To ensure privacy and confidentiality, the data was anonymized and aggregated at the point of collection, thereby removing any personal or identifying information. Our analysis begins on August 1, 2021, and ends on September 31, 2021. The Edge logs provide traffic information for roughly 10% of the desktop browsing activity in the United States^58^, accounting for millions of users. A common limitation of desktop browser-based studies of information consumption habits^20,45,48^ is they are unlikely to be representative of all American internet use^49^. Our focus is on comparing users’ consumption of and navigation to low-quality news sites, not on establishing precisely how much low-quality news is consumed by the average user. As long as any bias is consistent between news deserts and other areas, these comparisons remain informative about the difference in browsing habits. Further, after controlling for county population, there is no statistically significant correlation between a county being a news desert and its overall Edge usage.

To create our first set of measures, we record the number of requests for the sites in our lists of national news sites, low-quality information sites, and pink slime sites. For our second set of measures, we record the number of views of low-quality sites that were referred by either social media or search engines (per million referrals). Referrals are classified by capturing sequential visits within a 30 second time frame. We use the geographic information attached to the browsing data to create a daily county-level count of views for each type of site. For each county, we also collect the total amount of daily browser traffic. This number is used to re-scale our measures so that locations are more directly comparable. Each content type is normalized by the total requests for the county and then multiplied by one million.

### County-level control variables

Our demographic and other control variables were collected from OpenIntro. The repository (https://www.openintro.org/data/index.php?data=county_2019.) contains county-level information gathered by OpenIntro from the 2019 American Community Survey via the tidycensus R package. From the dataset, we use population (Population), Median household income (Median HHI), Percent of households that have broadband internet subscription (% Broadband), percentage of the population that is white (% White), and percent of the population 25 and older that earned a Bachelor’s degree or higher (% Bachelors Degree). Our final control, the county political leaning (% Republican), was included as past work^43–45,59^ has found that Republicans share and consume higher levels of low-quality news sources and was calculated from election data maintained by Harvard Dataverse at MIT Election Data + Science Lab (https://dataverse.harvard.edu/dataset.xhtml?persistentId=doi:10.7910/DVN/VOQCHQ.). The measure is the proportion of the county’s total votes that were cast for the Republican party nominee in the 2020 presidential election.

### Statistical analysis

The statistical analyses are conducted using OLS regression. For each model we include multiple specifications including adding control variables and fixed effects. Standard errors are clustered on the state-month. All analyses were conducted in R v3.6.3 and use the base stats package along with lfe 2.8-7.1.

## Supplementary Information

Supplementary Information.

## Data Availability

The datasets used and/or analysed during the current study are available from the corresponding author on reasonable request.
