# Supplementary material for: An evaluation of online information acquisition in US news deserts
Source: Sci Rep. 2024 Nov 13;14:27780. doi: 10.1038/s41598-024-77303-y (PMC11560965; doi:10.1038/s41598-024-77303-y)
Supplement: Supplementary file 1 — Supplementary Information. [file 41598_2024_77303_MOESM1_ESM.pdf]

Supplementary Materials for An Evaluation of Online Information Acquisition Habits in US News Deserts

*Kevin T. Greene et al.

*Corresponding author. Email: kg2082@princeton.edu

Contents

Descriptive Information **3**
Search Engines . . . 3
Social Media . . . 3
Sources of Low-Quality Domains . . . 4
Distribution of News Traffic Across News Domains . . . 5

Regression Tables - Main Results **5**

Alternative Measures of National News and Low-quality Domains **11**

Controlling for Sinclair TV Stations **12**

Regression Tables - Alternative News Desert Measure **15**

News Deserts and Activity **20**

Matching **21**

Descriptive Information

Content Types

Table S1 shows the descriptive statistics for the views across different newspaper content types. Table S2 reproduces Table S1 for the coarsened exact matched dataset.

Content	N	Mean	St. Dev.	Median
National News	189,877	26,294.490	17,247.030	24,397.970
Low-quality Content	189,877	1,629.910	2,643.777	1,258.596
Pink Slime	189,877	1.483	24.769	0.000

Table S1: Views across content types (per million requests)

Content	N	Mean	St. Dev.	Median
National News	154,320	26,600.750	17,126.610	24,725.840
Low-quality Content	154,320	1,667.586	2,749.818	1,245.680
Pink Slime	154,320	1.475	26.122	0.000

Table S2: Views across content types (per million requests) for the matched dataset

News Sites

Below are the 25 national news sites used for our analyses.

yahoo, cnn, nytimes, foxnews, msn, news.google, washingtonpost, finance.yahoo, usatoday, cnbc, news.yahoo, nypost, bbc, dailymail, politico, nbcnews, theguardian, businessinsider, thehill, huffpost, forbes, people, wsj, cbsnews, abcnews

Search Engines

Below are the search engines used for our analyses.

google, bing, duckduckgo, aol, yahoo, msn, baidu, ask, yandex, ecosia, startpage

Social Media

Below are the social media sites used for our analyses.

facebook, twitter, instagram, youtube, linkedin, pinterest, tiktok, reddit, nextdoor, myspace, whatsapp, weibo, quora, tumblr, flickr, vimeo, medium, digg, gab, rumble, bitchute, gettr, parler, truthsocial, 8kun, 4chan

Sources of Low-Quality Domains

The table below provides descriptive information about each of the sources of low-quality domains used in the main paper. We include the number of sites in each list, the industry of the list creator, and the criteria used to define low-quality sites.

Source	# Domains	Type	LQ Type
(*1*)	66	Academic	Misinformation
(*2*)	568	Academic	Misinformation
(*3*)	3427	Academic	Misinformation
MBFC	1189	Factcheck	Conspiracy

Table S3: Descriptive information for the four sources of low-quality information used in the study.

Most Viewed Low-Quality Sites

Below are the top ten most viewed low-quality sites in our analyses. In total, we use five sources that maintain lists of low-quality sites. For our analysis, we consider a site to be low-quality if it has been identified by at least two of these sources. Three of the lists come from academic literature on the subject. These include the sets of domains complied by (*1–3*). We also include two lists from fact-checking and media accountability groups. These include Media Bias/Fact Check and NewsGuard. Our final list contains more than 1300 low-quality domains in total.

Rank	Domain
1	dailymail.co.uk
2	drudgereport.com
3	breitbart.com
4	worldstarhiphop.com
5	thegatewaypundit.com
6	newsmax.com
7	bitchute.com
8	freerepublic.com
9	zerohedge.com
10	rawstory.com

Table S4: Top 10 low-quality sites by views

Distribution of News Traffic Across News Domains

In Figure S1 we present an empirical cumulative distribution function showing the distribution of the total traffic across news domains for desktop browsing in the United States. Data are collected using Similarweb. The total news web traffic is highly concentrated. The top 25 domains account for around 65% of total news traffic.

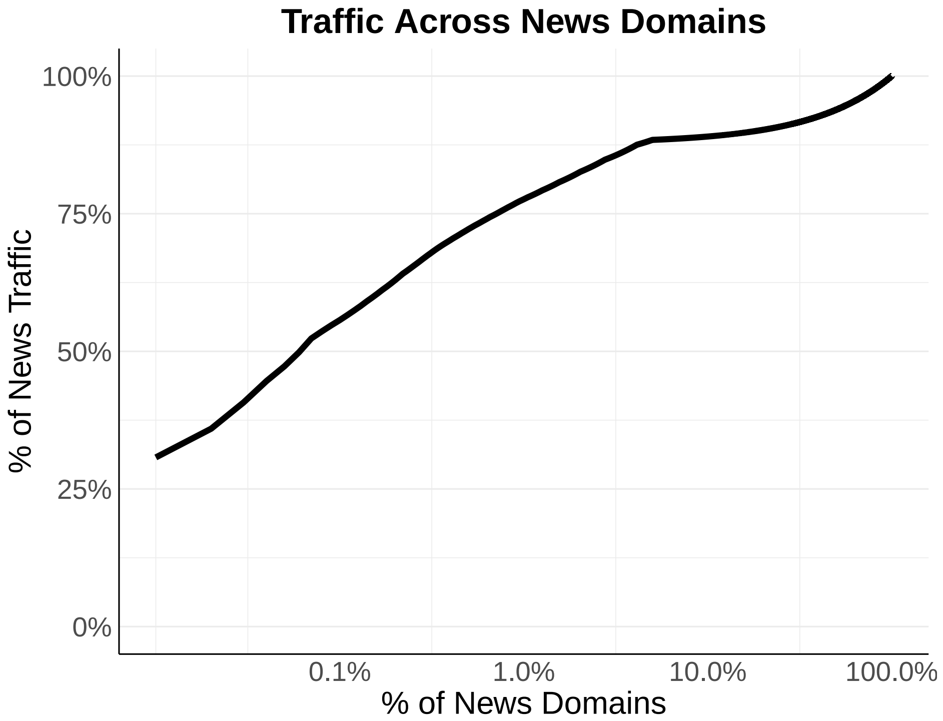

Figure S1: Concentration of traffic to news domains. Traffic measures are taken from Similarweb, and measure the share of total news traffic in the United States for each domain. Most news domains receive little traffic.

Regression Tables - Main Results

This section contains the full regression tables for the results in the main paper (Figures 4 and 5). In total, there are five tables including results for views of low-quality content, national news, pink slime, referrals to low-quality sites from search engines, and referrals to low-quality sites from social media.

	Dependent variable:		
	Low-Quality Views (per million)		
	M1	*M3*	*M2*
	(1)	(2)	(3)
News Desert	-18.220	-18.656	23.546
	(47.500)	(65.468)	(68.952)
Population		-0.0001^{***}	-0.001^{***}
		(0.00003)	(0.0003)
Med. Income		0.0001	-0.007
		(0.003)	(0.005)
% Broadband		6.876	12.062
		(3.344)	(5.441)
% White		3.788	2.821
		(3.313)	(3.224)
% B.A. Degree		4.345	17.358
		(4.652)	(9.073)
% Republican		2.700	1.020
		(3.412)	(3.835)
Constant	$1,639.848^{***}$		468.327^{***}
	(40.887)		(376.079)
Day FE		✓	
State FE		✓	
Matching			✓
Observations	189,877	188,230	153,283
Note:			*p<0.1; **p<0.05; ***p<0.01

Table S5: Regression Results, Low-Quality Views

	Dependent variable:		
	National News Views (per million)		
	M1	*M3*	*M2*
	(1)	(2)	(3)
News Desert	276.897	973.277***	331.214
	(373.041)	(252.176)	(341.161)
Population		−0.002***	−0.019***
		(0.0003)	(0.003)
Med. Income		0.001	0.019
		(0.014)	(0.026)
% Broadband		−76.891*	22.184
		(33.439)	(46.962)
% White		55.340**	87.891***
		(22.788)	(19.531)
% B.A. Degree		52.593	99.619**
		(35.651)	(33.541)
% Republican		34.997	−41.775
		(34.268)	(25.423)
Constant	26,143.460***		18,070.370***
	(349.463)		(3,335.938)
Day FE		✓	
State FE		✓	
Matching			✓
Observations	189,877	188,230	153,283
Note:			*p<0.1; **p<0.05; ***p<0.01

Table S6: Regression Results, National News

	Dependent variable:		
	Pink Slime Views (per million)		
	M1	*M3*	*M2*
	(1)	(2)	(3)
News Desert	-0.090	-0.091	−0.297
	(0.223)	(0.143)	(0.305)
Population		-0.00000	0.000**
		(0.00000)	(0.00000)
Med. Income		-0.00000	−0.00003
		(0.00001)	(0.00002)
% Broadband		0.055	0.024
		(0.034)	(0.026)
% White		-0.045	−0.024
		(0.023)	(0.021)
% B.A. Degree		-0.009	-0.008
		(0.017)	(0.016)
% Republican		-0.001	0.018
		(0.011)	(0.011)
Constant	1.532***		2.364
	(0.255)		(1.664)
Day FE		✓	
State FE		✓	
Matching			✓
Observations	189,877	188,230	153,283
Note:	*p<0.1; **p<0.05; ***p<0.01		

Table S7: Regression Results, Pink Slime

	Dependent variable:		
	Referrals to Low-Quality Sites from Search		
	M1	*M3*	*M2*
	(1)	(2)	(3)
News Desert	−6.339	-6.243	-2.475
	(5.425)	(6.250)	(6.041)
Population		−0.00001***	−0.0001**
		(0.00000)	(0.00002)
Med. Income		0.0001	−0.001**
		(0.0003)	(0.0003)
% Broadband		-0.261	0.712
		(0.545)	(0.664)
% White		0.437*	0.582**
		(0.229)	(0.257)
% B.A. Degree		0.278	1.231*
		(0.436)	(0.638)
% Republican		0.069	0.032
		(0.369)	(0.376)
Constant	140.337***		50.514
	(3.470)		(44.713)
Day FE		✓	
State FE		✓	
Matching			✓
Observations	189,877	188,230	153,283
Note:		*p<0.1; **p<0.05; ***p<0.01	

Table S8: Regression Results, Search Engine Referrals

	Dependent variable:		
	Referrals to Low-Quality Sites from Social Media		
	M1	*M3*	*M2*
	(1)	(2)	(3)
News Desert	-1.372^{**}	-0.872	-0.872
	(0.667)	(0.722)	(0.787)
Population		-0.00000^{***}	-0.00001^{**}
		(0.00000)	(0.00000)
Med. Income		-0.0001^{***}	-0.0001^{***}
		(0.00004)	(0.00004)
% Broadband		0.271^{***}	0.382^{***}
		(0.057)	(0.082)
% White		-0.106	-0.059
		(0.085)	(0.054)
% B.A. Degree		0.161^{*}	0.155
		(0.085)	(0.138)
% Republican		0.191^{***}	0.080
		(0.070)	(0.050)
Constant	15.257^{***}		-9.566^{**}
	(0.529)		(4.038)
Day FE		✓	
State FE		✓	
Matching			✓
Observations	189,877	188,230	153,283
Note:			*p<0.1; **p<0.05; ***p<0.01

Table S9: Regression Results, Social Media Referrals

Alternative Measures of National News and Low-quality Domains

In Figure S6 we reproduce our main results after excluding dailymail, bbc, theguardian and news.google.com. While the first three are highly trafficked domains in the US, as they are UK based news sites they may be conceptually different than the other sites in our list of news sites. Second, news.google.com does not produce original reporting, instead serving as a portal to other news sources. This alternative measure produces results that are consistent with those presented in the main paper.

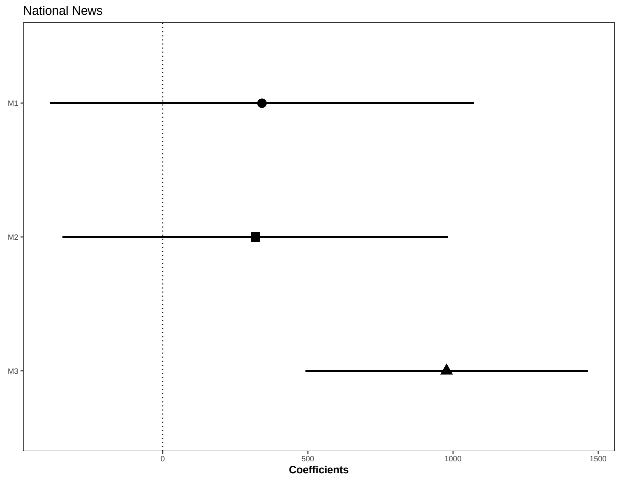

Figure S2: Coefficients and 95% confidence intervals for the impact of news deserts on consumption habits. (A) views of low-quality news (B) views of national news (C) views of pink slime. In each figure M1 indicates the bivariate correlation, M2 includes control variables and coarsened exact matching, and M3 includes control variables and fixed effects.

In Figure S3 we reproduce our main results after excluding bitchute from our list of low-quality domains. While this is highly trafficked domain in the US, it is a video sharing platform which is different than the other sites in our list. This alternative measure produces results that are consistent with those presented in the main paper.

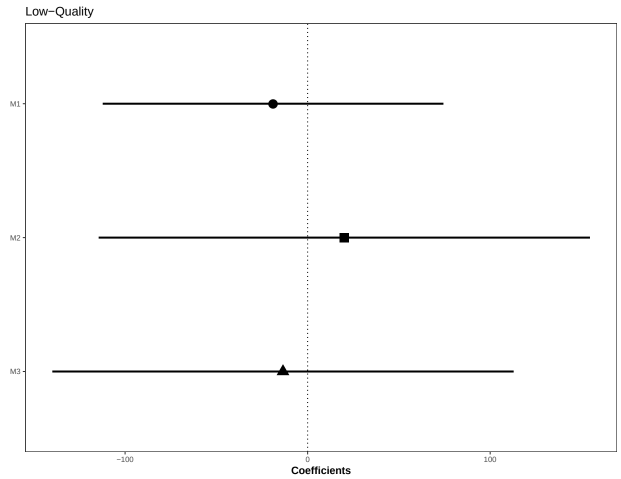

Figure S3: Coefficients and 95% confidence intervals for the impact of news deserts on consumption habits. (A) views of low-quality news (B) views of national news (C) views of pink slime. In each figure M1 indicates the bivariate correlation, M2 includes control variables and coarsened exact matching, and M3 includes control variables and fixed effects.

Controlling for Sinclair TV Stations

In this section, we re-estimate the models from the main paper, now including a variable measuring if a Sinclair news station is present in the county. (*4*, *5*) have previous found that the ownership of local TV stations impacts political attitudes.

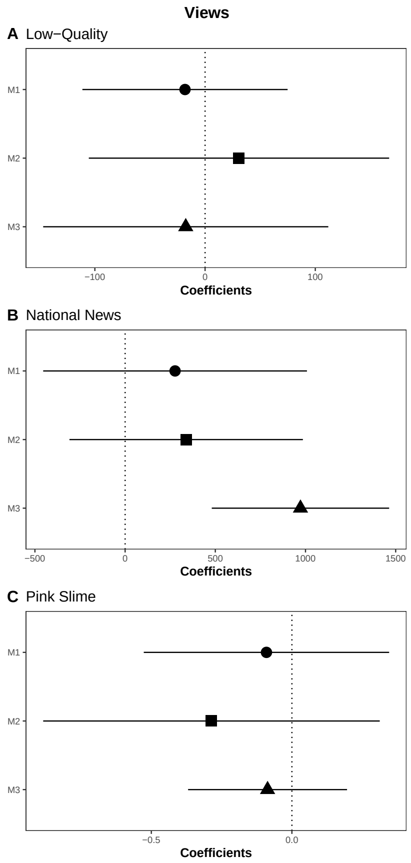

Figure S4: Coefficients and 95% confidence intervals for the impact of news deserts on consumption habits. (A) views of low-quality news (B) views of national news (C) views of pink slime. In each figure M1 indicates the bivariate correlation, M2 includes control variables and coarsened exact matching, and M3 includes control variables and fixed effects.

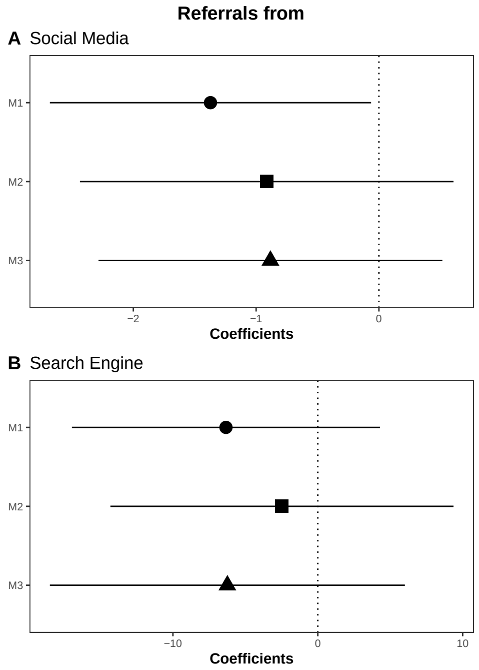

Figure S5: Coefficients and 95% confidence intervals for the impact of news deserts on referrals to misinformation sites. (A) referrals from social media (B) referrals from search engines (C) views of pink slime. In each figure M1 indicates the bivariate correlation, M2 includes control variables and coarsened exact matching, and M3 includes control variables and fixed effects

Regression Tables - Alternative News Desert Measure

In this section, we re-estimate the results in the main paper (Tables 4 and 5) using an alternative measure of news deserts. This measure considers a county to be a news desert if it has no local paper. In contrast, in the main paper, we considered a county to be a news desert if it had only a single paper. The findings using this stricter definition of news deserts are consistent with those in the main paper. We still see no evidence that news deserts consume more low-quality content.

	Dependent variable:		
	Low-Quality Views (per million)		
	M1	*M3*	*M2*
	(1)	(2)	(3)
News Desert	-248.522^{***}	-274.357^{**}	23.546
	(108.845)	(110.864)	(68.952)
Population		-0.0001^{***}	-0.001^{***}
		(0.00003)	(0.0003)
Med. Income		0.0002	-0.007
		(0.003)	(0.005)
% Broadband		5.996^{*}	12.062^{**}
		(3.371)	(5.441)
% White		3.590	2.821
		(3.254)	(3.224)
% B.A. Degree		4.356	17.358^{*}
		(4.671)	(9.073)
% Republican		2.791	1.020
		(3.441)	(3.835)
Constant	$1,640.885^{***}$		468.327
	(33.736)		(376.079)
Day FE		✓	
State FE		✓	
Matching			✓
Observations	189,877	188,230	86,378
Note:			*p<0.1; **p<0.05; ***p<0.01

Table S10: Regression Results, Low-Quality Views

	Dependent variable:		
	National News Views (per million)		
	M1	*M3*	*M2*
	(1)	(2)	(3)
News Desert	332.866	330.229	331.214
	(836.114)	(914.399)	(341.161)
Population		-0.002^{***}	-0.019^{***}
		(0.0003)	(0.003)
Med. Income		0.001	0.019
		(0.015)	(0.026)
% Broadband		-83.613^{**}	22.184
		(34.317)	(46.962)
% White		53.428^{**}	87.891^{***}
		(22.472)	(19.531)
% B.A. Degree		52.108	99.619^{***}
		(35.790)	(33.541)
% Republican		39.567	-41.775
		(34.136)	(25.423)
Constant	$26,279.790^{***}$		$18,070.370^{***}$
	(364.014)		(3,335.938)
Day FE		✓	
State FE		✓	
Matching			✓
Observations	189,877	188,230	86,378
Note:			*p<0.1; **p<0.05; ***p<0.01

Table S11: Regression Results, National News Views

	Dependent variable:		
	Pink Slime Views (per million)		
	M1	*M3*	*M2*
	(1)	(2)	(3)
News Desert	-0.510	0.002	-0.297
	(0.311)	(0.370)	(0.305)
Population		-0.00000	0.00000***
		(0.00000)	(0.00000)
Med. Income		-0.00000	−0.00003*
		(0.00001)	(0.00002)
% Broadband		0.056	0.024
		(0.034)	(0.026)
% White		−0.045**	-0.024
		(0.023)	(0.021)
% B.A. Degree		-0.009	-0.008
		(0.017)	(0.016)
% Republican		-0.001	0.018
		(0.011)	(0.011)
Constant	1.505***		2.364
	(0.270)		(1.664)
Day FE		✓	
State FE		✓	
Matching			✓
Observations	189,877	188,230	86,378
Note:			*p<0.1; **p<0.05; ***p<0.01

Table S12: Regression Results, Pink Slime Views

	Dependent variable:		
	Referrals to Low-Quality Sites from Search		
	M1	*M3*	*M2*
	(1)	(2)	(3)
News Desert	-42.135^{***}	-45.735^{***}	-2.475
	(10.765)	(9.751)	(6.041)
Population		-0.00001^{***}	-0.0001^{**}
		(0.00000)	(0.00002)
Med. Income		0.0001	-0.001^{**}
		(0.0003)	(0.0003)
% Broadband		-0.382	0.712
		(0.535)	(0.664)
% White		0.411^{*}	0.582^{**}
		(0.231)	(0.257)
% B.A. Degree		0.281	1.231^{*}
		(0.431)	(0.638)
% Republican		0.069	0.032
		(0.379)	(0.376)
Constant	138.741^{***}		50.514
	(3.269)		(44.713)
Day FE		✓	
State FE		✓	
Matching			✓
Observations	189,877	188,230	86,378
Note:		*p<0.1; **p<0.05; ***p<0.01	

Table S13: Regression Results, Search Engine Referrals

	Dependent variable:		
	Referrals to Low-Quality Sites from Social Media		
	M1	*M3*	*M2*
	(1)	(2)	(3)
News Desert	0.412	2.687	-0.872
	(4.769)	(5.575)	(0.787)
Population		-0.00000^{***}	-0.00001^{**}
		(0.00000)	(0.00000)
Med. Income		-0.0001^{***}	-0.0001^{***}
		(0.00004)	(0.00004)
% Broadband		0.288^{***}	0.382^{***}
		(0.047)	(0.082)
% White		-0.101	-0.059
		(0.087)	(0.054)
% B.A. Degree		0.162^{*}	0.155
		(0.085)	(0.138)
% Republican		0.185^{***}	0.080
		(0.071)	(0.050)
Constant	14.491^{***}		-9.566^{**}
	(0.326)		(4.038)
Day FE		✓	
State FE		✓	
Matching			✓
Observations	189,877	188,230	86,378
Note:			*p<0.1; **p<0.05; ***p<0.01

Table S14: Regression Results, Social Media Referrals

News Deserts and Activity

We assess the correlation between overall browsing activity and a location being a news desert. We find that locations with larger populations and a higher percent of the population with access to broadband are associated with increase online activity. We do not find a statistically significant relationship between a location being a news desert and online activity.

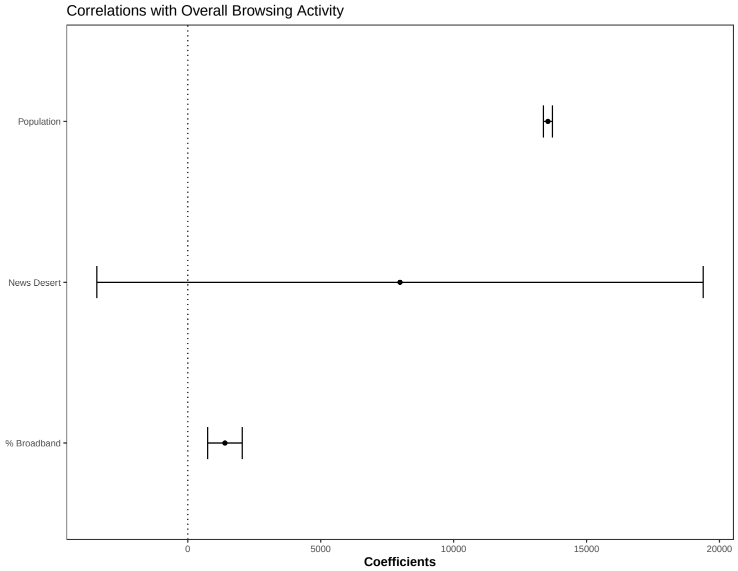

Figure S6: Coefficients and 95% confidence intervals for the correlations with overall activity. The unit of analyses is the county.

Matching

Past work has found that news deserts are more likely to occur in locations that are smaller, less educated, less wealthy, and with less access to broadband internet (*6*, *7*). These differences make direct comparisons between news deserts and locations with local media more difficult. We use coarsened exact matching, which allows us to match news deserts with non-news deserts that have similar characteristics. The matching implements the recommendations by (*8*) and uses the MatchIt package in R (*9*).

Before matching, the relevant variables mentioned previously have large standard mean differences (SMD) across the treatment and control groups. As expected from past work, news deserts, and non-news deserts have very different characteristics. We use the coarsened exact matching algorithm to correct these imbalances. Under the hood, coarsened matching, as opposed to exact matching, obviates the need to exclude many observations from the dataset by binning numerical values to reduce the level of granularity in the data before performing matching. After matching, the absolute SMDs for all our variables are near .1, in line with current best practices. We also use visual diagnostics to confirm that after matching, news deserts, and non-news deserts are similar to each other across features.

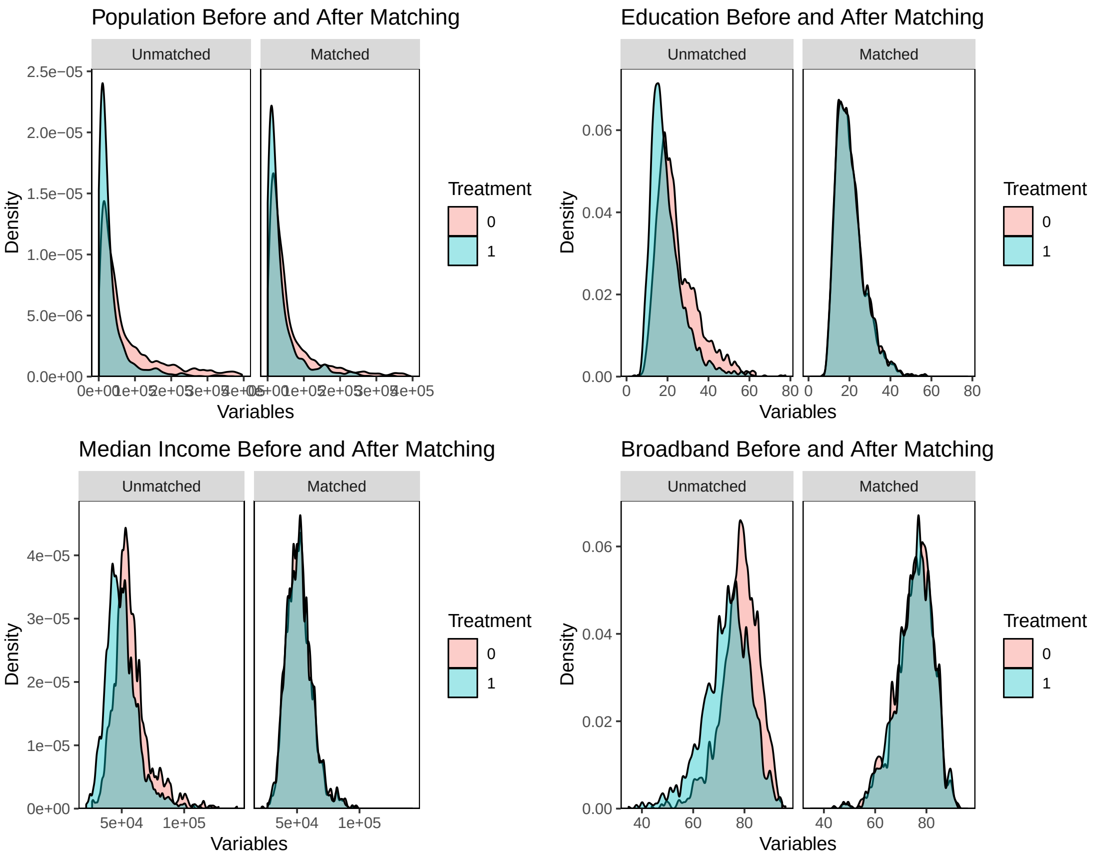

Figure S7: The distribution of variables in the treatment and control groups before and after matching.

References

1. G. Pennycook, D. G. Rand, *Proceedings of the National Academy of Sciences* **116**, 2521–2526 (2019).
2. W. Chen, D. Pacheco, K.-C. Yang, F. Menczer, *Nature communications* **12**, 1–10 (2021).
3. A. Hounsel *et al.*, presented at the 10th {USENIX} Workshop on Free and Open Communications on the Internet ({FOCI} 20).
4. G. J. Martin, J. McCrain, *American Political Science Review* **113**, 372–384 (2019).
5. M. S. Levendusky, *Political Communication* **39**, 23–38 (2022).
6. P. M. Abernathy, *The expanding news desert* (Center for Innovation and Sustainability in Local Media, 2018).
7. P. M. Abernathy, *News deserts and ghost newspapers: Will local news survive?* (University of North Carolina Press, 2020).
8. D. E. Ho, K. Imai, G. King, E. A. Stuart, *Political analysis* **15**, 199–236 (2007).
9. E. A. Stuart, G. King, K. Imai, D. Ho, *Journal of statistical software* (2011).
